# Our Bureau of Information

**Published:** 1912-01-20

**Authors:** 


### OUR BUREAU OF INFORMATION.

### How the Work Grows—What we are Doing and Where Help Can be Given.

Although our Bureau is as yet only in its initial stages, we have been able to help many of those who have applied to us for advice or assistance. Not all, because there are so many people who, reading an application, are content to say to themselves "What a sad case this is!" without asking themselves if they can help to relieve it, either personally or by making some appeal to others. Perhaps they have an idea, such as "I wonder if So-and-So could not help!" or "Perhaps the case would be suitable for such a place!" but the thought is never carried into action. This slackness arises from two causes, of which one is a sort of moral laziness and the other the conviction—only too deeply ingrained in the British mind—that those who cannot give money can give nothing. We wish our readers would believe that we welcome every well-meant suggestion. It may not fit the particular case for which it is intended, but may be the very thing for some other which we have in hand or which may come before us in the future. Last week we appealed for cases to be sent to us. This week we appeal for information about places all over the country. We are prepared to verify any recommendations given and to file them for reference. And to this end we want more correspondents. We have now correspondents in Rochdale, Seaford, and Wandsworth, in addition to those who have previously been mentioned, but we require many more.

Let us state what these workers have done, as it may give a hint to others. One has made inquiries about the social position of an applicant, which will aid us in suggesting the proper sort of institution for his case; for it must be remembered that it is not only the poor who are sometimes at a loss how to deal with special cases of ill-health or distress, and our advice must be regulated by a knowledge of the patient's and applicant's environment. Another was able to visit a case and tell the family where the help they needed was to be found. This was a case in his own neighbourhood, but he was able also to give information as to the best methods of dealing with another one in a different locality. To another we are sending for information as to a home the proprietor of which seeks to have placed on our Approved List. In all these cases our correspondents—members of the Brotherhood of Social Service—have given no money, but they have given time and thought, and the benefit of their local knowledge and personal experience. Another friend—an honorary member of the Brotherhood—has given a ticket for a convalescent home for a case that was sent to us. This will give temporary relief—very gratefully accepted—while we find something of permanent benefit for a most deserving individual. He has given money, not personal service. And what we want our readers to understand is that both are needed. No one is too poor to help us, for we want other things than money. No one is too busy to help us, for we want other things than time. We want everything that anyone who loves his fellow-men has to give.

It is impossible for the editor of a newspaper which is published in London to deal from personal knowledge with every case that is brought before him. If we are told that someone in Manchester is suffering from a disease that would benefit by salt-water baths, is it any good to tell him that there is a sea-bathing hospital at Margate? He wants something near at hand, but as yet we have no one to tell us what he can get at Southport or Blackpool. We are asking for cases that need help from all over the kingdom, and to meet these we need advisers and correspondents all over the kingdom.

In questions of information, as apart from actual help, we need still more to have far-reaching advice. Let us illustrate by an instance which has already passed through our hands. A query came to us from Ireland; the answer to it came from Newcastle-on-Tyne. Without the Bureau our Irish friend would still have been groping, uncertain what to do. With it, the advice required was easily obtained. Sometimes, it is true, we can give the information required from the office, or at least indicate where it can be obtained, but this is by no means an invariable rule, and it is obvious that it cannot be certain in matters pertaining to local institutions. For that we must depend on local helpers. Therefore we ask those, far and near, who wish to help the poor and suffering, to assist us. Let them tell us what they want and what they can do, and send us their grievances and their grumbles. We have a considerable amount of knowledge at our back already; we want more, and can never have too much. Those we cannot help at the moment we shall certainly do our best to help so soon as is humanly possible. But our readers must help us.

### Needs and Helpers.

Earning a Livelihood while Undergoing a Cure.

"Oxymoron" writes: Could you or any of your readers kindly tell me what chance, if any, there is for a young man of twenty, with incipient phthisis, to earn his living while leading a sanatorium life? He has had a good business training, and is fond of farm pursuits. Do not some sanatorium superintendents take men like this as secretaries, or in some capacity connected with the institution?

("R. M. O." answers: "Oxymoron" might write to the Coppins Green Sanatorium, Milnathort, Kinross-shire, N.B., for the treatment of early tuberculosis in men of limited means. Patients are employed at graduated work under medical supervision in the gardens and nursery, and are paid for all work done. They are thereby enabled to reduce the cost of treatment to themselves, and at the same time gain experience in a suitable occupation. The terms for treatment are 30s. a week, less the wages earned.)

Medico-Psychological Certificate.

"Newcastle" writes: Can you tell me if the certificate of the Medico-Psychological Association, necessary for male nurses, can be obtained by correspondence?

(The only persons eligible for the examination of the Medico-Psychological Association are those who have served for three years in an asylum for the insane, including the probationary period of three or six months. Instruction is given in the asylum, if it is a recognised training school. An asylum with 100 beds is usually a recognised mental training school.)

Home Wanted for Epileptic Lady.

"M. W." writes: Can you assist me in finding a home for an epileptic lady, aged about 48, at from 10s. to 12s. weekly. I have tried for years to find an institution suitable, but without success. At present she resides in private families, but constantly has to leave, and should be under proper care if only it were possible to find a place at the low price named.

Nurses for Mission Hospitals.

"E. B. F." writes: A friend of mine, who is a medical missionary in India, has written to say that for her hospital (100 beds) new sisters are required. To get these should one advertise in the ordinary nursing journals, or does there exist a special missionary journal for nurses?

(Advertise in the *Nursing Mirror*, or write to the Hospital for Women and Children, 7 Lupus Street, S.W., which is in connection with the Zenana Medical College, or to the Medical Mission Hospital, Balaam Street, Plaistow, E.)

